# Features of steroidogenesis in men with hypogonadism and type 2 diabetes

**DOI:** 10.14341/probl13129

**Published:** 2022-06-16

**Authors:** R. V. Rozhivanov, M. O. Chernova, V. A. Ioutsi, G. A. Mel’nichenko, M. V. Shestakova, N. G. Mokrysheva

**Affiliations:** Endocrinology Research Centre; Endocrinology Research Centre; Endocrinology Research Centre; Endocrinology Research Centre; Endocrinology Research Centre; Endocrinology Research Centre

**Keywords:** hypogonadism, men, testosterone, diabetes mellitus, mass-spectrometry, steroidogenesis

## Abstract

**BACKGROUND:**

BACKGROUND: Type 2 diabetes mellitus (DM2) in men is associated with a high incidence of hypogonadism. Testosterone is a steroid hormone and one of the final metabolites of steroidogenesis, which causes interest in assessing the content of key steroid hormones, their precursors and metabolites in hypogonadal and eugonadal men with T2DM.

**AIMS:**

AIMS: Assessment of the features of steroidogenesis in men with hypogonadism in T2DM using tandem mass spectrometry.

**MATERIALS AND METHODS:**

MATERIALS AND METHODS: A full-design, cross-sectional, screening, single-center, non-interventional study included men with T2DM, who were he was treated in Endocrinology Research Centre, Moscow. The study was conducted from October 2021 to January 2022. Medical history assessment, physical examination with determination of body mass index (BMI), measurement of key steroid hormones, their precursors and metabolites by isotope dilution liquid chromatography/tandem mass spectrometry, glycated hemoglobin (HbA1c) were performed. The groups were compared using the Mann-Whitney U-test for quantitative indicators and χ² with Yates’ correction for qualitative ones. Correlation analysis was performed by the Spearman correlation method. When determining the criterion of statistical significance, the Bonferroni correction was applied.

**RESULTS:**

RESULTS: Patients with hypogonadism had statistically significantly more pronounced obesity compared with eugonadal men. In a comparative analysis of patients, depending on the presence of hypogonadism, there were statistically significantly lower levels of androgen precursors 17-hydroxypregnenolone and 17-hydroxyprogesterone in hypogonadal men. At the same time, a positive statistically significant correlation was found between total testosterone and 17-hydroxyprogesterone. In addition, 17-hydroxyprogesterone, although to a lesser extent, but positively correlated with other androgens - androstenedione (r=0,328; p<0,001) and dehydroepiandrosterone (r=0,183; p=0,004). >< 0,001) and dehydroepiandrosterone (r=0,183; p=0,004).

**CONCLUSIONS:**

CONCLUSIONS: In this investigation the prevalence of male hypogonadism in type 2 diabetes, determined by high-precision tandem mass spectrometry, was 69,5%. There was no effect of the disease on the mineralocorticoid and glucocorticoid links of adrenal steroidogenesis. Hypogonadism was associated with decreased levels of a number of testosterone precursors. The most significant of them was 17-hydroxyprogesterone, which can be considered as a marker of testicular steroidogenesis.

## Abstract (ru)

**ОБОСНОВАНИЕ:**

ОБОСНОВАНИЕ. Сахарный диабет 2 типа (СД2) у мужчин ассоциирован с высокой частотой встречаемости синдрома гипогонадизма. Тестостерон является стероидным гормоном и одним из итоговых метаболитов стероидогенеза, что обуславливает интерес к оценке содержания ключевых стероидных гормонов, их предшественников и метаболитов у мужчин с СД2 в условиях гипо- и эугонадизма.

**ЦЕЛЬ:**

ЦЕЛЬ. Оценка особенностей стероидогенеза у мужчин с гипогонадизмом при СД2 с использованием тандемной масс-спектрометрии.

**МЕТОДЫ:**

МЕТОДЫ. В сплошное одномоментное скрининговое одноцентровое неинтервенционное исследование были включены мужчины с СД2, обратившиеся за медицинской помощью в ФГБУ «НМИЦ эндокринологии» МЗ РФ в период с октября 2021 по январь 2022 г. Проводились изучение анамнеза, физикальный осмотр с определением индекса массы тела, гликированного гемоглобина, измерение ключевых стероидных гормонов, их предшественников и метаболитов методом тандемной масс-спектрометрии. Сравнение групп проведено с помощью U-критерия Манна–Уитни и χ² с поправкой Йейтса. Корреляционный анализ выполнен методом Спирмена. При определении критерия статистической значимости применялась поправка Бонферрони.

**РЕЗУЛЬТАТЫ:**

РЕЗУЛЬТАТЫ. Пациенты с гипогонадизмом имели статистически значимо более выраженное ожирение по сравнению с мужчинами без гипогонадизма. При сравнительном анализе пациентов в зависимости от наличия гипогонадизма отмечались статистически значимо более низкие показатели предшественников андрогенов 17-гидроксипрегненолона и 17-гидроксипрогестерона у мужчин в группе гипогонадизма. При этом была выявлена положительная статистически значимая корреляция между общим тестостероном и 17-гидроксипрогестероном. Кроме того, 17-гидроксипрогестерон хоть и в меньшей силе, но положительно коррелировал с другими андрогенами — андростендионом (r=0,328; p<0,001) и дегидроэпиандростероном (r=0,183; p=0,004). ЗАКЛЮЧЕНИЕ. В нашем исследовании частота встречаемости гипогонадизма у мужчин с СД2 при определении высокоточным методом тандемной масс-спектрометрии составила 69,5%. Не отмечено влияния заболевания на минералокортикоидное и глюкокортикоидное звенья надпочечникового стероидогенеза. Гипогонадизм ассоциировался со снижением уровня ряда предшественников тестостерона. Наиболее значимым из них являлся 17-гидроксипрогестерон, который может рассматриваться как маркер тестикулярного стероидогенеза. >< 0,001) и дегидроэпиандростероном (r=0,183; p=0,004).

## RELEVANCE

Many studies have reported development of hypogonadism in males with type 2 diabetes (T2D) [1–3]. T2D males have lower testosterone compared to healthy ones (by an average of 2.5 nmol/L), whereas incidence of hypogonadism in T2D males is higher than inthose without diabetes; according to different studies, this incidence may exceed 50% [4–6]. A 2017–2018 study involving a Russian population of males with T2D established that the incidence of hypogonadism amounted to 32,7% [7]. The reason hypogonadism develops is that in T2D, metabolic syndrome and obesity cause a disruption of negative feedback loop between pituitary gland and gonads, thus leading to lower release of testosterone [4, 8]. Testosterone is a steroid hormone and one of the end metabolites ofsteroidogenesis [9][10]. This explains the relevance of studying the levels of key steroid hormones, their precursors and their metabolites in T2D males. However, such analysis is difficult as most of laboratory methods are not precise enough [11–13]. This is why tandem mass spectrometry is a preferred method of determining the levels of steroidogenesis components, as this technology provides higher sensitivity and specificity [13–15].

## OBJECTIVE

Analyse the specifics of steroidogenesis in males with hypogonadism and T2D by using tandem mass spectrometry.

## METHODS

## Research design

A full-design, cross-sectional, screening, single-centre, non-interventional study.

## Acceptance criteria

Inclusion criteria: males 40–65, T2D confirmed as per the algorithms in place at the time of study [16].

Non-inclusion criteria: sex and developmental disorders; adrenal pathology (including adrenal diseases in medical history); a missing testicle or both testicles; cryptorchism; injuries of or surgeries on genitals; administration of androgens, anabolic steroids, gonadotropins, antioestrogens or antiandrogens, whether at the time of study or in medical history; alcohol or drug addiction.

Exclusion criteria: turning down the offer to take part in the study; exiting the study programme.

## Participants

Males with T2D who were treated in Endocrinology Research Centre (Moscow) between October 2021 and January 2022 were included in the study.

## Study duration

Research material was collected from October 2021 to January 2022.

## Medical intervention

Blood samples were taken in the mornings (7 to 11 a.m.) in fasting state from median cubital veins.

## Key result

Analysis of specifics of steroid profile in T2D males in relation to hypogonadism.

## Additional results

Analysis of the correlation between reduced testosterone and the release of other steroid hormones in T2D males.

## Subgroup analysis

Patient group with hypogonadism was compared to that without it.

## Results registration method

Medical history data were obtained through a questionnaire and analysis of the subjects’ data retrieved from the qMS healthcare information system (run by National Endocrinology Research Centre). Physical examination included observation of pubic hair, breast glands and genitals. Total testosterone, cortisol, cortisone, 21-deoxycortisol, 11-deoxycortisol, aldosterone, corticosterone, 11-deoxycorticosterone, pregnenolone, progesterone, 17hydroxypregnenolone, 17-hydroxyprogesterone, dehydroepiandrosterone, and androstenedione were measured by high-performance liquid chromatography with tandem mass spectrometry (HPLC-MS/MS) with Agilent 1290 Infinity II chromatograph and AB Sciex TripleQuad 5500 mass spectrograph. Glycated haemoglobin was measured by charge-transfer high-performance liquid chromatography (HPLC) with BIO-RAD D10 automatic analyser. Hypogonadism was determined when total testosterone in blood serum was under 12.1 nmol/L.

Diabetes complications were diagnosed as per the algorithms in place at the time of the study [16].

## Ethical review

Research protocol of the study entitled “Mass Spectrometry Diagnostics and Personalisation of Hypogonadism Treatment in Males with Type 2 Diabetes” was approved by Endocrinology Research Centre’s internal Ethics Committee on 13 October 2021 (official session record no. 21) subject to Ordinance 311 issued on 12 August 2021 by the Ministry of Health introducing the regulations concerning access to and use of intellectual property represented by Endocrinology Research Centre’s endocrinopathy databases and/or data retrieved therefrom. This article presents a fragment of the non-interventional part of the research.

## Statistical analysis

Sample size determination principles: sample size was determined based on an expected incidence of 25%, no-show ratio of 20% and 10% width of the 95% confidence interval.

Statistical analysis methods: raw data were processed with a set of STATISTICA applications (StatSoft Inc., USA, version 8.0). Quantitative data are presented as a median and interquartile range; qualitative data are presented as percentage values. For comparison between groups, a non-parametric method χ² with Yates correction was used for qualitative data and the Mann-Whitney U-test for quantitative data. Spearman correlation method was applied in correlation analysis. Since our intergroup and correlation analysis of steroid profile involved multiple comparison and verification of 13 hypotheses, the Bonferroni correction was used to reassess the p value. Any difference with a p value under 0.0042 was considered significant.

## FINDINGS

## Study subjects (participants)

This study included 347 males with T2D. Sample properties are presented in Table 1.

**Table:** Table 1. General characteristics of the sample of patients Notes: quantitative data are presented as a median and interquartile range; qualitative data are presented as percentage values. T2D — type 2 diabetes; BMI — body mass index; HbA1c  — glycated haemoglobin; DPP4i — dipeptidyl peptidase IV inhibitors; SGLT2i — sodium-glucose cotransporter 2 inhibitors; GLP-1RAs — glucagon-like peptide-1 receptor agonists; SU — sulphonylurea drugs.

Age (years)	59 [ 53; 62]
T2D duration (years)	12 [ 6; 17]
BMI (kg/m2)	32.2 [ 28.7; 35.9]
HbA1c (%)	8.6 [ 7.2; 10.1]
Antihyperglycemic therapy (%)
Metformin	59.4
DPP4i	19.3
SGLT2i	21.6
GLP-1RAs	7.5
Sulphonylurea	27.4
Combined therapy, including long-acting insulin alternatives	11.5
Basal bolus therapy	34.3
Insulin therapy (total)	45.8
T2D complications (%)
Retinopathy	43.2
Nephropathy	27.4
Polyneuropathy	67.7
Coronary heart disease	26.2
Acute myocardial infarction in medical history	5.2
Acute Cerebrovascular Event in medical history	1.2
Diabetic foot disease (all forms)	31.7
No complications found	14.1
Steroid profile (nmol/L)
Total testosterone	10.3 [ 7.5; 13.0]
Cortisol	330 [ 260; 422]
Cortisone	50.1 [ 42.0; 58.4]
21-deoxycortisol	0.026 [ 0.010; 0.100]
11-deoxycortisol	0.76 [ 0.48; 1.30]
Aldosterone	171 [ 105; 280]
Corticosterone	4.74 [ 2.90; 8.25]
11-deoxycorticosterone	0.05 [ 0.01; 0.10]
Pregnenolone	1.50 [ 1.00; 2.21]
Progesterone	<0.1 [ <0.1; 0.1]
17-hydroxypregnenolone	2.0 [ 1.32; 3.30]
17-hydroxyprogesterone	1.34 [ 0.99; 1.90]
Dehydroepiandrosterone	5.10 [ 2.97; 8.10]
Androstenedione	2.40 [ 1.84; 3.10]

## Key findings

Comparative analysis of patients in relation to hypogonadism revealed significantly reduced levels of androgen precursors 17-hydroxypregnenolone and 17-hydroxyprogesterone in males within the hypogonadism group (see Table 2).

**Table:** Table 2. Comparison between patients in relation to hypogonadism status Notes: *the Mann-Whitney U-test’ **χ² with Yates correction. Quantitative data are presented as a median and interquartile range; qualitative data are presented as percentage values. The Bonferroni correction was applied, p<0,0042. T2D — type 2 diabetes; BMI — body mass index; HbA1c — glycated haemoglobin; DPP4i — dipeptidyl peptidase IV inhibitors; SGLT2i — sodium-glucose cotransporter 2 inhibitors; GLP-1RAs — glucagon-like peptide-1 receptor agonists; SU — sulphonylurea drugs.

Age (years)	59 [ 54; 62]	59 [ 52; 62]	0.654*
T2D duration (years)	12 [ 6; 18]	11 [ 6; 15]	0.284*
BMI (kg/m2)	33.1 [ 29.0; 37.3]	30.8 [ 27.7; 33.5]	<0.001*
HbA1c (%)	8.8 [ 7.5; 10.3]	8.1 [ 6.7; 9.5]	0.005*
Antihyperglycemic therapy (%)
Metformin	62.2	52.8	0.127**
DPP4i	15.4	28.3	0.008**
SGLT2i	23.2	17.9	0.334**
GLP-1RAs	6.6	9.4	0.491**
Sulphonylurea	29.1	23.6	0.358**
Combined therapy, including long-acting insulin alternatives	12.5	9.4	0.530**
Basal bolus therapy	32.8	37.7	0.440**
Insulin therapy (total)	45.2	47.2	0.828*
T2D complications (%)
Retinopathy	42.7	44.3	0.873**
Nephropathy	26.6	29.3	0.699**
Polyneuropathy	68.1	67.0	0.943**
Coronary heart disease	26.6	25.5	0.937**
Acute myocardial infarction in medical history	5.0	5.7	0.999**
Acute Cerebrovascular Event in medical history	1.7	0.0	0.318**
Diabetic foot disease (all forms)	35.3	23.6	0.042**
No complications found	14.9	12.3	0.623**
Steroid profile (nmol/L)
Total testosterone	8.6 [ 6.8; 10.6]	15.0 [ 13.3; 17.7]	<0.001*
Cortisol	335 [ 260; 431]	324 [ 260; 400]	0.386*
Cortisone	50.0 [ 41.7; 58.0]	52.1 [ 44.5; 59.7]	0.214*
21-deoxycortisol	0.03 [ 0.01; 0.10]	0.03 [ 0.01; 0.10]	0.503*
11-deoxycortisol	0.75 [ 0.49; 1.30]	0.76 [ 0.44; 1.29]	0.819*
Aldosterone	180 [ 110; 292]	166 [ 100; 258]	0.269*
Corticosterone	4.7 [ 2.8; 8.0]	5.1 [ 3.1; 8.5]	0.427*
11-Deoxycorticosterone	0.05 [ 0.01; 0.10]	0.07 [ 0.03; 0.11]	0.015*
Pregnenolone	1.45 [ 0.98; 2.17]	1.71 [ 1.20; 2.33]	0.018*
Progesterone	<0.10 [ <0.10; 0.10]	0.10 [ <0.10; 0.13]	0.019*
17-hydroxypregnenolone	1.87 [ 1.20; 2.99]	2.19 [ 1.50; 3.80]	0.004*
17-hydroxyprogesterone	1.20 [ 0.90; 1.67]	1.74 [ 1.33; 2.40]	<0.001*
Dehydroepiandrosterone	5.0 [ 2.9; 7.6]	5.6 [ 3.2; 8.4]	0.146*
Androstenedione	2.34 [ 1.79; 2.97]	2.60 [ 1.93; 3.41]	0.008*

Moreover, patients with hypogonadism had significantly greater degree of obesity than those without it.

## Additional findings

In order to assess the correlation between testosterone level and the release of other steroids in T2D males, a correlation analysis was carried out (see Table 3).

**Table:** Table 3. Correlation analysis results Notes: Spearman correlation method was applied. Bonferroni correction was applied, p<0,0042.

Parameter	r	p
Total testosterone & cortisol	-0.048	0.454
Total testosterone & aldosterone	0.061	0.342
Total testosterone & cortisone	0.126	0.051
Total testosterone & 21-deoxycortisol	-0.027	0.673
Total testosterone & 11-deoxycortisol	-0.039	0.543
Total testosterone & 17-hydroxyprogesterone	0.406	<0.001
Total testosterone & 17-hydroxypregnenolone	0.152	0.018
Total testosterone & corticosterone	-0.023	0.719
Total testosterone & 11-deoxycorticosterone	0.039	0.546
Total testosterone & progesterone	0.141	0.028
Total testosterone & pregnenolone	0.099	0.122
Total testosterone & androstenedione	0.175	0.006
Total testosterone & dehydroepiandrosterone	0.098	0.127

We established a significant positive correlation between total testosterone and 17hydroxyprogesterone. Moreover, 17-hydroxyprogesterone, although to a lesser degree, did positively correlate with other androgens: androstenedione (r=0.328; p<0.001) and dehydroepiandrosterone (r=0.183; p=0.004).

No adverse developments were registered

## DISCUSSION

## Summary of key findings

Reduced level of testosterone in hypogonadism and T2D is associated with a reduction of 17hydroxyprogesterone which, in turn, is associated with reduced levels of other androgens.

## Discussion of key findings

Uneven distribution of incidence of hypogonadism in T2D males is well known: this incidence varies from 15% to 80% [4][6][7][17][18]. This is explained by differences in the degree of carbohydrate metabolism compensation, the degree of obesity, patient age, methods used to measure blood testosterone, and diagnosis criteria applied [13][16][19]. The association of hypogonadism with obesity which we established has been confirmed by many different studies and can be explained by a functional disruption of the negative feedback loop in the hypothalamic-hypophyseal-testicular axis [4][8][20–22]. This disruption may be caused by different factors.

Some early studies established an unusually low release of gonadotropins after administration of gonadotropin-releasing hormone in males with T2D and obesity [23]. Moreover, hypogonadism may develop due to resistance of the central parts of hypothalamus-pituitary axis to leptin in obesity and also due to excess levels of oestrogens building up in adipose tissue, which disrupts the release of gonadotropin-releasing hormone and gonadotropins [24][25]. A functional suppression of regulatory hormones due to theaforementioned factors causes a reduction of their stimulation impact on steroidogenesis in Leydig cells. Thus, testicular steroidogenesis is suppressed, whereas the release of steroid hormones in adrenal glands remains unaffected. The results of our study support this hypothesis, since we found no abnormalities in the mineralocorticoid and glucocorticoid steps of adrenal steroidogenesis in males with T2D, obesity and hypogonadism or eugonadism.

However, some studies describe the impact of insulin resistance and obesity in terms of increased levels of aldosterone and cortisol [26][27]; therefore, further studies are needed to establish the impact of carbohydrate metabolism disorder on adrenal steroidogenesis.

Luteinising hormone interacting with G protein-coupled receptor and triggering a chain of reactions involving eventual activation of protein kinase A is the key regulator of testicular steroidogenesis in Leydig cells [9][10]. Protein kinase A is a trigger of several intracellular processes resulting in cholesterol transfer from lipid droplets into mitochondria and an increasing release of genes coding the ferments of testicular steroidogenesis [28–30]. Introduction of chorionic gonadotropin, which is similar to luteinising hormone, caused the release of mRNA regulatory genes of testicular steroidogenesis in the treated cells to rise several times higher than control values [29].

At present, studies are underway into intracellular signal pathways and additional mechanisms of testicular steroidogenesis; however, such inquiries are difficult due to low sensitivity and specificity of existing methods [28][30–32]. Given a similar chemical structure ofkey steroid hormones, their precursors and their metabolites, and the difficulties of differentiating between them through standard immunoenzymometric methods of diagnostics [11–13], HPLC-MS/MS is currently the optimal method of analysing the components ofsteroidogenesis, as it provides virtually 100% selectivity, an ample sensitivity and repeatability [13–15]. Thus, in a 2021 PIVUS study conducted in Sweden with 452 healthy-appearing males aged 70, HPLC-MS/MS was used to measure their steroid profile (pregnenolone, 17hydroxypregnenolone, 17­hydroxyprogesterone, 11-deoxycortisol dehydroepiandrosterone, androstenedione, testosterone, oestrone, and estradiol); the study confirmed an association between low testosterone and obesity: significantly lower levels oftestosterone and its precursors (17hydroxypregnenolone, 17­hydroxyprogesterone, androstenedione, and dehydroepiandrosterone) were found in males with obesity compared to those with normal body weight and overweight [33].

In our study, within the T2D group low testosterone was also associated with higher BMI and coincided with lower levels of androgen precursors, i.e., 17hydroxypregnenolone and 17hydroxyprogesterone. An additional correlation analysis demonstrated a most significant positive correlation between the levels of testosterone and 17hydroxyprogesterone, whereas the latter, although to a lesser degree, did positively correlate with weaker androgens, i.e., androstenedione and dehydroepiandrosterone. Thus, one may surmise that 17hydroxyprogesterone is a marker of testicular steroidogenesis.

Our assumption is aligned with findings of several studies [34][35]. These reports have demonstrated a positive correlation between intratesticular testosterone and serum 17hydroxyprogesterone in males after administration of chorionic gonadotropin [34]. Based onthis observation, the authors proposed that serum 17hydroxyprogesterone could be used as a market of intratesticular production of testosterone in males. To verify this hypothesis, a prospective study was conducted, in which 140 males were divided into 3 groups: those receiving stimulation therapy with clomiphene citrate and/or human chorionic gonadotropin; those receiving exogenous replacement therapy with testosterone drugs (TRT) to suppress intratesticular production of testosterone; and fertile males with regular serum testosterone levels as a control group.

During the study, testosterone level was within the normal range, while 17-hydroxyprogesterone significantly reduced in patients receiving TRT and significantly increased in those receiving stimulation therapy with clomiphene/gonadotropin. Based on this, the authors concluded that serum 17-hydroxyprogesterone is a reliable marker of intratesticular production of testosterone and thus can be used for titration/replacement of drugs affecting steroidogenesis in Leydig cells [34]. Thus, in recent clinical tests of a new intranasal form oftestosterone, which the authors positioned as a kind of TRT with minimum suppressing impact on the hypothalamic-hypophyseal-testicular axis due to short exposure time, normal level of 17-hydroxyprogesterone during therapy was considered as a marker of preserved testicular steroidogenesis, minimum risk of spermatogenesis suppression and possibility to continue the therapy with short acting testosterone. Reduced 17-hydroxyprogesterone indicated a suppressive effect of the therapy [35]. Since the alternative way of determining intratesticular production of testosterone is an invasive biopsy of testes, establishing a marker enabling to determine it by analysing a blood sample goes a long way towards streamlining the diagnostics.

## Limitations of this study

The sample group was made of patients who were treated in a large federal clinic; thus, specifics of steroidogenesis we have determined may vary in the general population of T2D males, especially given the impact of the degree of obesity on steroidogenesis parameters. Moreover, data obtained through tandem mass spectrometry cannot be fully extrapolated to other methods of measuring steroid levels in males.

## CONCLUSION

In our study, the incidence of hypogonadism in T2D males was determined through high-precision tandem mass spectrometry and amounted to 69.5%. Development of hypogonadism in T2D males was associated with obesity. No significant impact of the disease on the mineralocorticoid and glucocorticoid steps of adrenal steroidogenesis was identified. Hypogonadism is associated with a reduced level of several testosterone precursors. The most significant of them is 17-hydroxyprogesterone which can be considered as a marker oftesticular steroidogenesis.

## ADDITIONAL INFORMATION

Funding source. This study was funded by Besins Healthcare RUS.

Conflict of interest. Roman V. Rozhivanov: in 2017–2022, Roman V. Rozhivanov was remunerated by Besins Healthcare RUS for his educational lectures; Mariia O. Chernova: no conflict of interest; Vitaliy A. Ioutsi: no conflict of interest; Galina A. Mel’nichenko: in 2017–2022, Galina A. Mel’nichenko was remunerated by Besins Healthcare RUS for her educational lectures; Marina V. Shestakova: no conflict of interest; Natalya G. Mokrysheva: no conflict of interest.

Authors’ contribution. Roman V. Rozhivanov: study concept, academic material collection and processing, article drafting; Mariia O. Chernova: academic material collection and processing, article drafting; Vitaliy A. Ioutsi: academic material collection; Galina A. Mel’nichenko: study concept, article editing; Marina V. Shestakova: study concept, article editing; Natalya G. Mokrysheva: article editing.

Acknowledgments. The authors express their gratitude to all patients who took part in this study.
